# Supplementary material for: Identification of a Golgi GPI-N-acetylgalactosamine transferase with tandem transmembrane regions in the catalytic domain
Source: Nat Commun. 2018 Jan 26;9:405. doi: 10.1038/s41467-017-02799-0 (PMC5785973; doi:10.1038/s41467-017-02799-0)
Supplement: Supplementary file 1 — Supplementary Information [file 41467_2017_2799_MOESM1_ESM.docx]

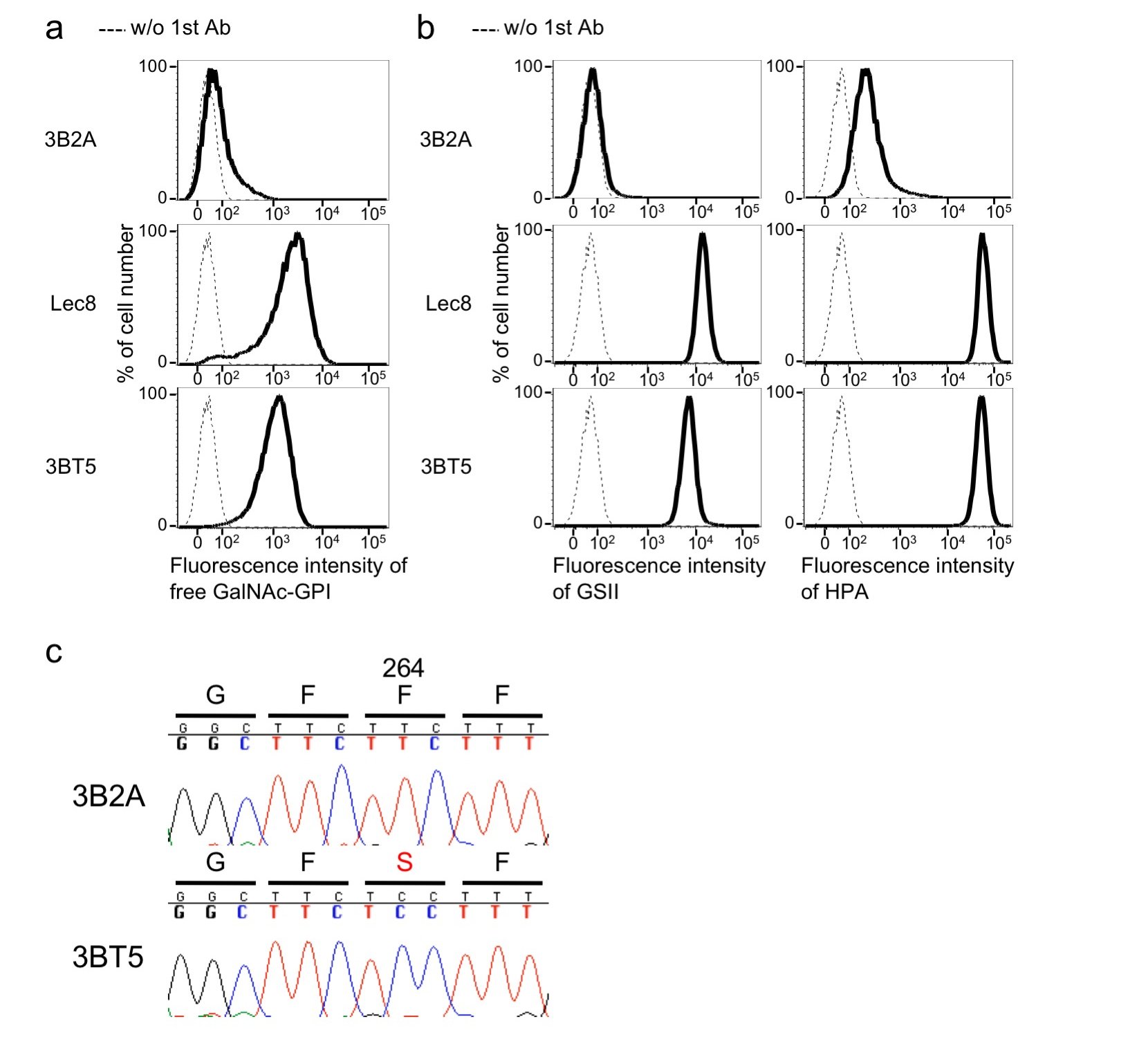


Supplementary Figure 1. Characterization of 3BT5 cells derived from 3B2A CHO cells, related to Fig. 1. **a** T5 mAb staining of 3BT5 cells. Dotted lines, background staining by secondary antibody (rat monoclonal anti-mIgM) only. Representative results from two independent experiments. **b** Surface glycan profiles of 3BT5 cells. Cells were stained by fluorescent-GSII and -HPA lectins (binding to non-reducing terminal GlcNAc and GalNAc, respectively). Representative results from two independent experiments. **c** *SLC35A2* gene mutation in 3BT5 cells. *SLC35A2* cDNA from 3B2A and 3BT5 cells were sequenced by Sanger method. Missense mutation causing F264S was identified in 3BT5 cells.


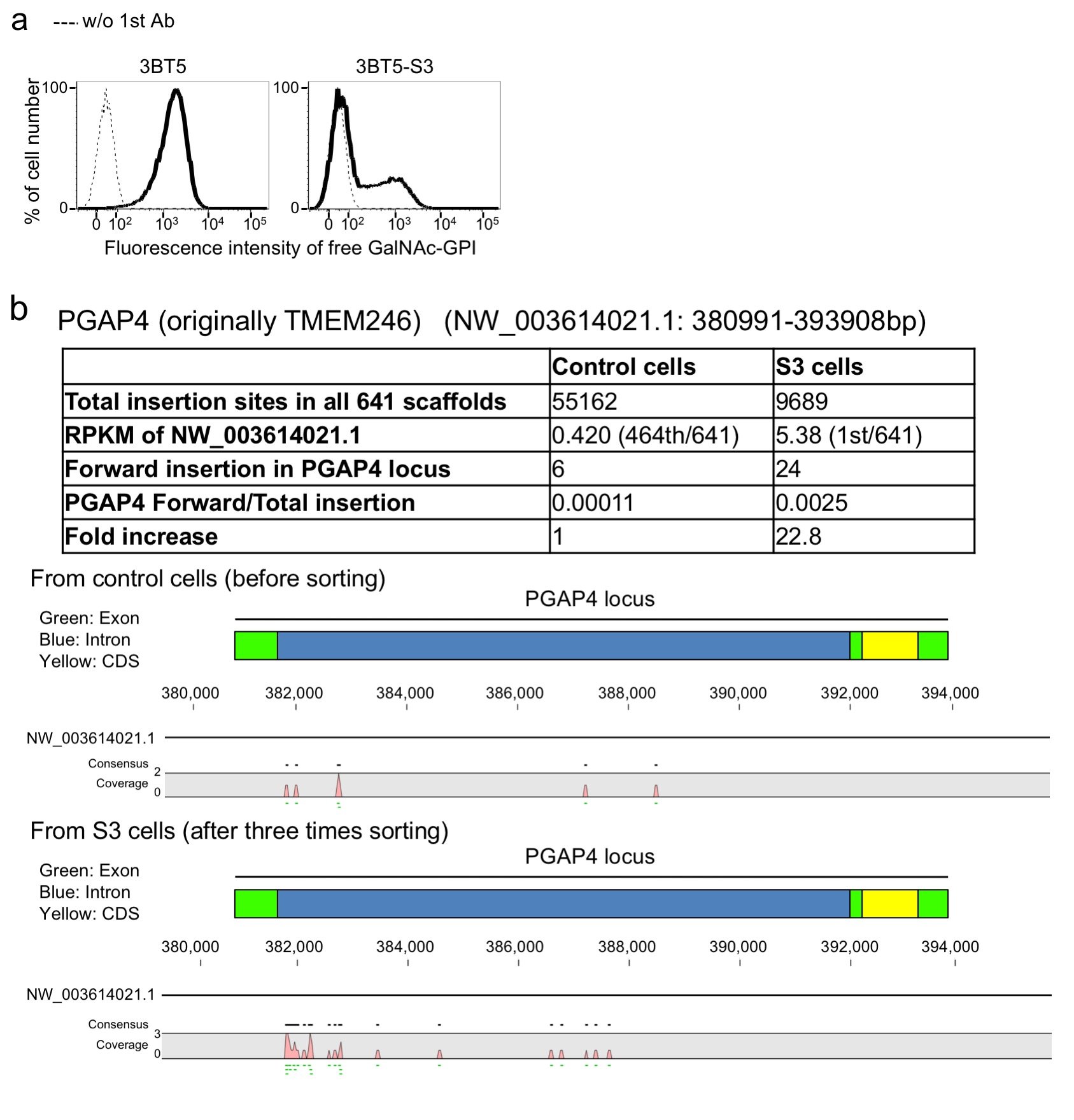


Supplementary Figure 2. Forward genetic screening of GPI-GalNAc transferase, related to Fig. 2. **a** T5 mAb staining of gene-trapped mutant cells. The staining intensity by T5 mAb was analyzed in gene-trapped mutant cells after third round of sorting (3BT5-S3) to monitor enrichment of target mutant population. Dotted lines indicated background staining. Rat monoclonal anti-mIgM was used as a secondary antibody. **b** Results of next generation sequencing. Upper table shows total insertion sites, Reads Per Kilobase of exon per Million mapped reads (RPKM) score, forward insertion sites in PGAP4 locus, ratio of forward insertion sites to total insertion sites, and the fold increase of the ratio of forward insertion sites to total insertion sites in S3 cells relative to control cells. Lower panel shows forward insertion sites in PGAP4 locus. Control and S3 cells, gene-trapped 3BT5 cells before sorting and after three rounds of sorting, respectively. Green, blue, and yellow regions indicate exon, intron, and CDS in PGAP4 locus, respectively.


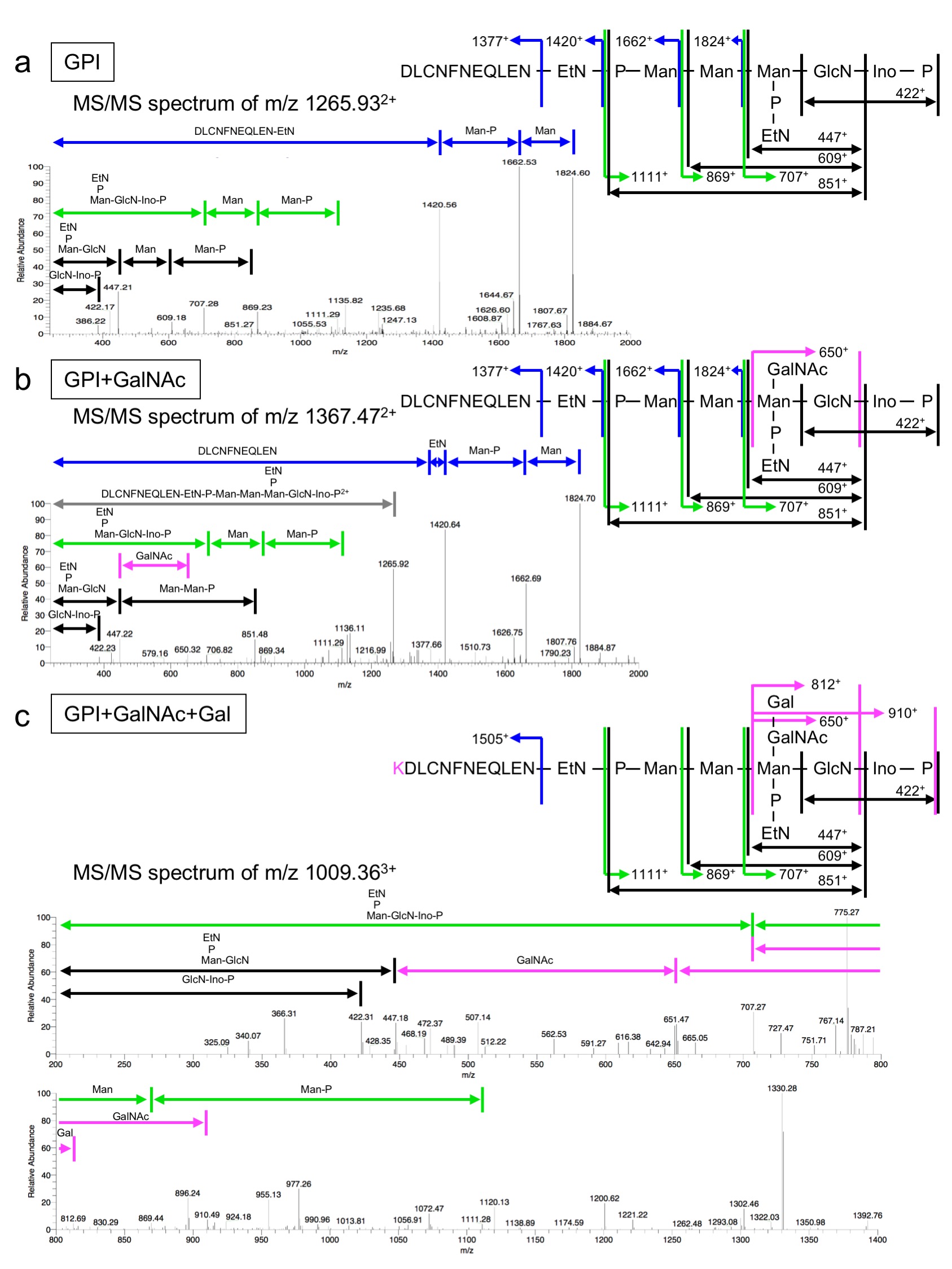


Supplementary Figure 3. ESI-MS/MS analysis of GPI in purified CD59, related to Fig. 2 and 7. **a**-**c** MS/MS spectra of C-terminal peptide with GPI cleaved by PI-PLC were displayed. **a**, 1265.93^2+^ for GPI core only; **b**, 1367.47^2+^ for GPI core + GalNAc; **c**, 1009.36^3+^ for GPI core + GalNAc + Gal with an additional lysine residue.


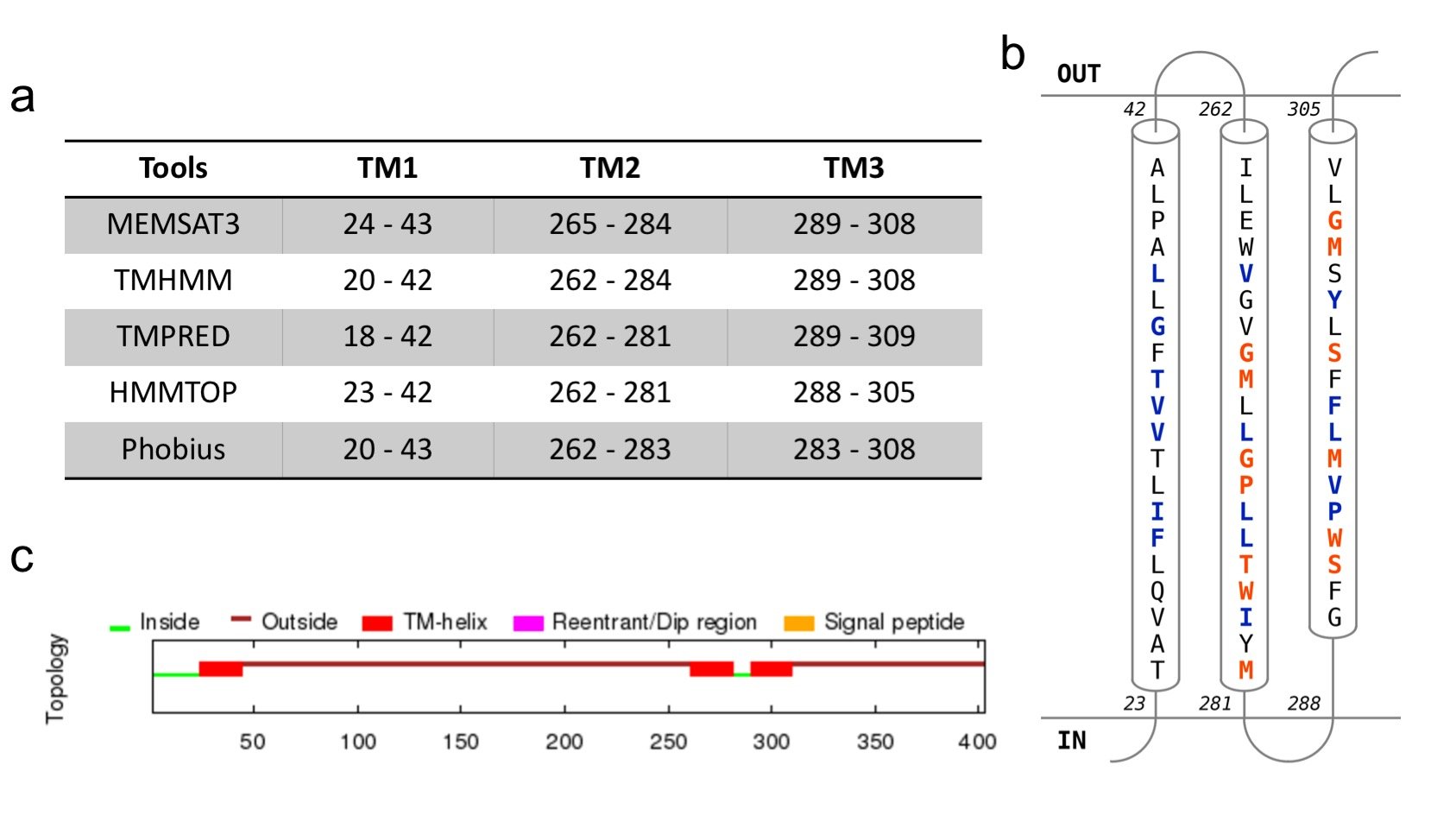


Supplementary Figure 4. In silico analysis of PGAP4, related to Fig. 3. **a** Table of compiled results of prediction of PGAP4 topology. Table indicates tools used and the TMDs predicted by each software. **b** and **c**. Topology of PGAP4 predicted by computational approach.


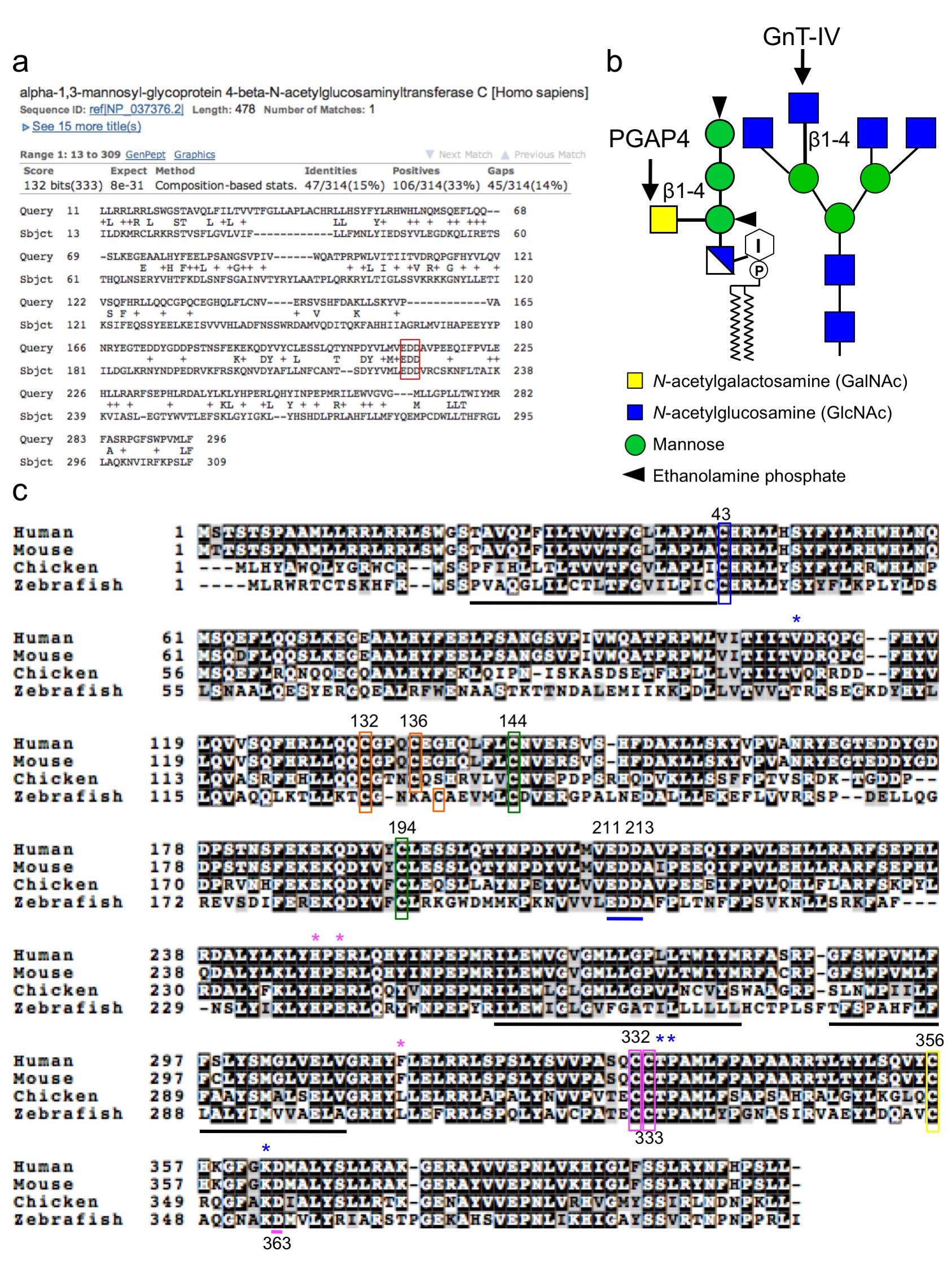


Supplementary Figure 5. Homology search by PSI-BLAST program and alignment of PGAP4 amino acid sequences, related to Fig. 3, 4, 5, and 6. **a** PSI-BLAST search against non-redundant protein sequence database using human PGAP4 sequence as a query. GnT-IV-A, B, and C were identified, in which GnT-IV-C most frequently appeared so that the sequence alignment was presented. **b** Schematic of the reactions of PGAP4 and GnT-IV. GnT-IV transfers GlcNAc to mannose of *N*-glycan in a β-1, 4 linkage. PGAP4 transfers GalNAc to mannose of GPI in a β-1, 4 linkage. Thus, GnT-IV and PGAP4 use mannose and UDP-HexNAc as acceptor and donor substrates, respectively, having functional similarity. **c** Alignment of vertebrate PGAP4 sequences. Sequence alignment was constructed using ClustalW and BoxShade. Residues conserved among species were shaded in gray or black. Black underlines indicate transmembrane domains. Blue and red underlines are a DXD-like motif and a catalytic site, respectively. Conserved cysteines are surrounded by colored boxes in that the same colors indicate the pairing in disulfide bonds. Free cysteines are indicated in blue and yellow boxes. Residues related to binding UDP-GalNAc and GPI-glycan are indicated in blue and red asterisks, respectively. PGAP4 sequences are from NCBI; human (NP_115718.1); mouse (NP_080220.2); chicken (XP_004949556.1); zebrafish (NP_001070809.1).


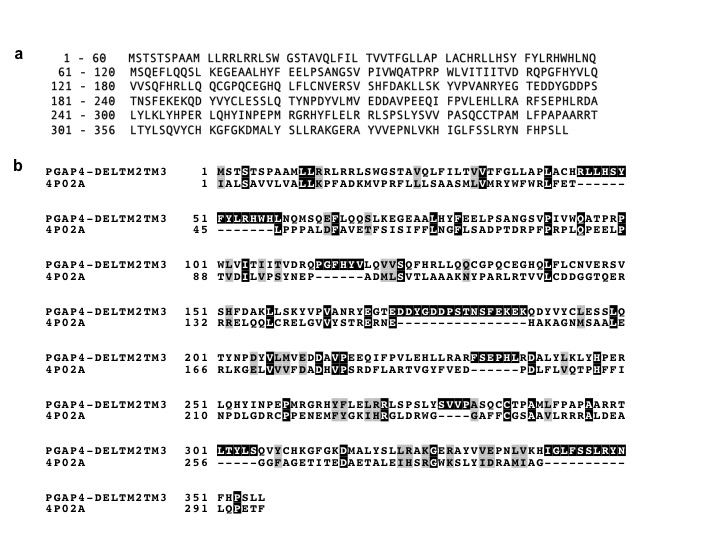


Supplementary Figure 6. Amino acid sequence of PGAP4ΔTM2, TM3 used for 3D modeling (**a**) and alignment with cellulose synthase GT-A domain (**b**), related to Fig. 4. PGAP4ΔTM2, TM3 lacks 47 amino acids (262-308) corresponding to TM2 and TM3. Amino acid numbers after 262 are shifted by 47 because of the deletion.


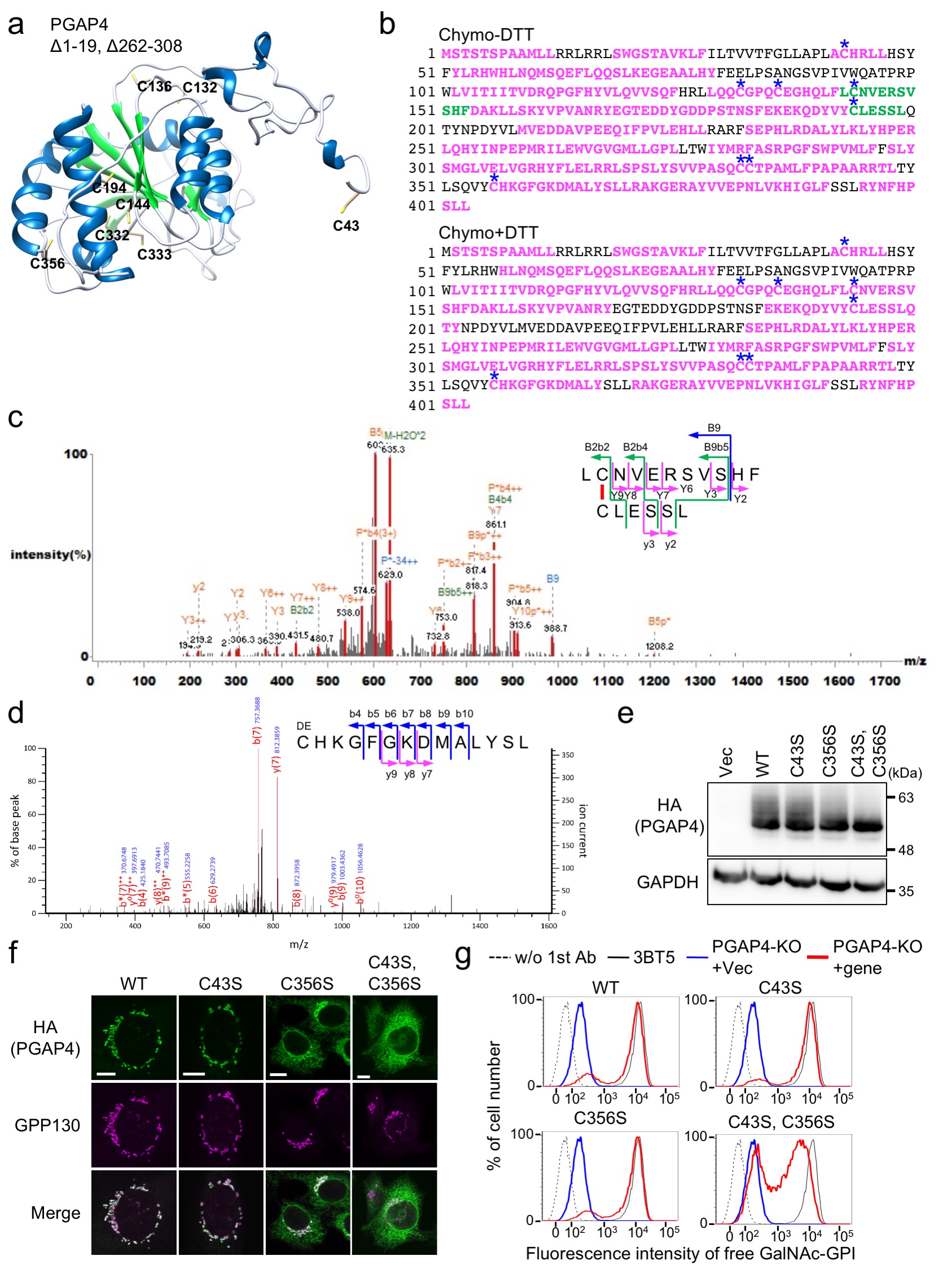


Supplementary Figure 7. Determination of disulfide bonds in PGAP4, related to Fig. 4. **a** 3D structural model of PGAP4Δ1-19, Δ262-308 is displayed in ribbon model. **b** 3FLAG-hPGAP4(Q26K)-3HA was purified and run on SDS-PAGE under non-reducing conditions. PGAP4 was digested by chymotrypsin with or without DTT followed by LC-ESI-MS/MS analysis. Data was analyzed using MASCOT database and an algorithm DBond for identification of disulfide-linked peptides. Detected fragments are indicated in magenta and green. Cysteines are indicated by blue asterisks. Fragments containing C43 or C356 were detected with and without DTT, indicating no disulfide bond between them. A disulfide-linked adduct containing C144 and C194 was detected without DTT (colored in green), indicating existence of a disulfide bond (See **c** and **d** for the MS/MS profiles). Fragments containing C132 and C136, and C332 and C333 were detected under both conditions while these fragments were dehydrogenized under non-reducing conditions, indicating existence of disulfide bonds. **c** MS/MS profile of peptides linked via a disulfide bond between C144 and C194. **d** MS/MS profile of a peptide containing C356 detected without DTT. DE: Dehydrogen. **e**-**g**. Mutation analysis of C43 and C356 residues. **e** Western blotting of mutants (C43S, C356S, and C43S, C356S-double mutant). **f** Immunofluorescence imagining of mutants. GPP130, the Golgi marker. Scale bars: 10 µm. **g** Functional analysis of mutants by transfection into PGAP4-KO cells and flow cytometry for restoration of free GPI expression. Black, blue, red, and dotted lines indicate 3BT5 cells, PGAP4-KO cells transfected with empty vector, PGAP4-KO cells transfected with mutant PGAP4 in pTK plasmid, and background staining, respectively. Cells were stained by T5 mAb and goat polyclonal anti-mIgM as a secondary antibody. Representative data from two independent experiments are shown. **d**-**g** indicated that C43 and C356 are not linked by a disulfide bridge.

Supplementary Figure 8. 3D structural model of PGAP4, related to Fig. 4. **a** Full length PGAP4 composed of GT-A core and transmembrane region. N- and C-terminals are indicated. **b** DXD-like motif (E211-D212-D213) and the catalytic residue D363 are highlighted in sphere model. **c** Putative GPI binding site formed by H247, E249 and F313.


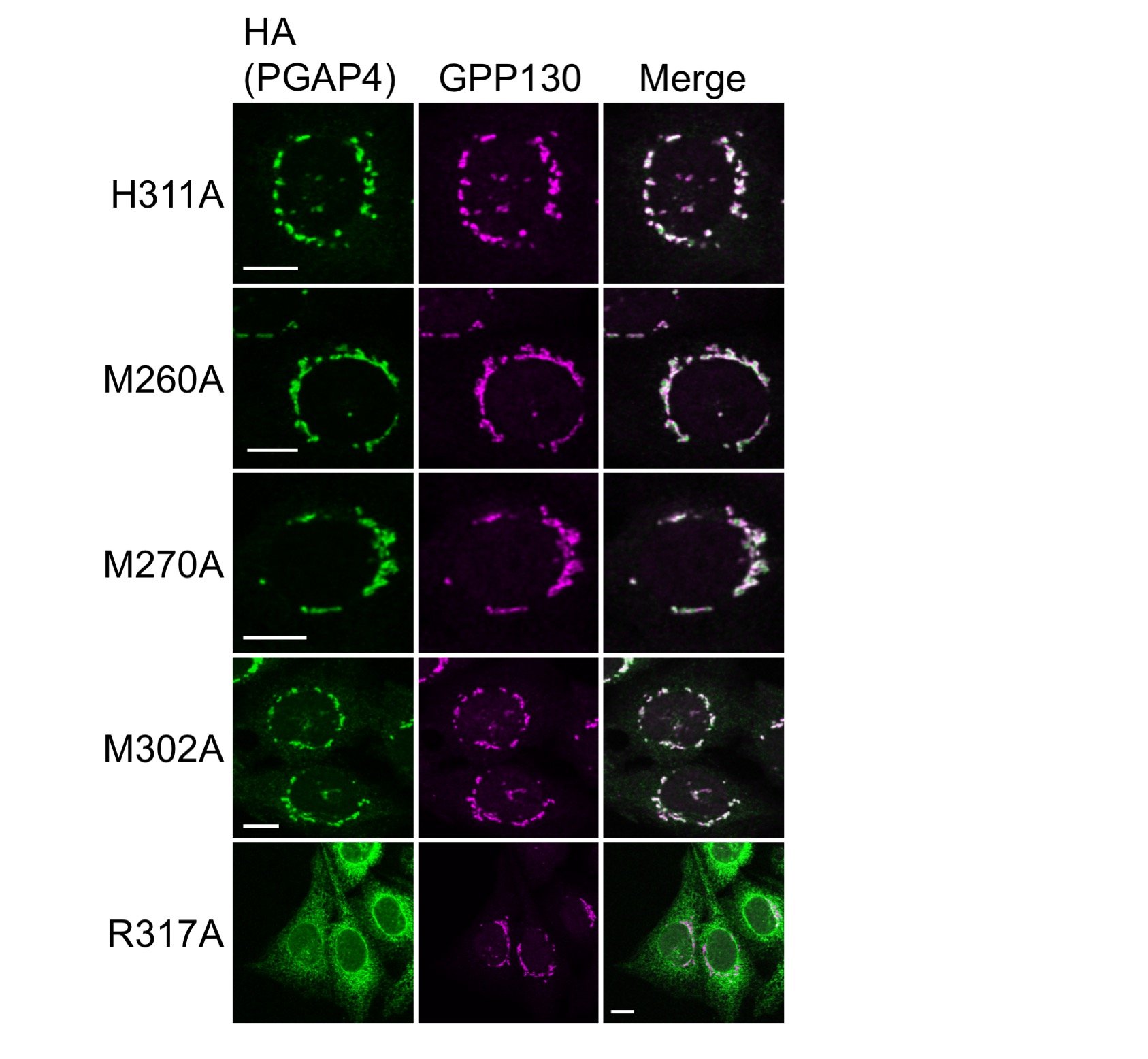


Supplementary Figure 9. Subcellular localization of PGAP4 mutants, related to Fig. 5 and 6. 3BT5-PGAP4-KO cells were transiently transfected with pME-hPGAP4-3HA. GPP130, the Golgi marker. All mutants except R317A were localized at the Golgi. R317A mutant was accumulated in the ER. Scale bars: 10 µm.


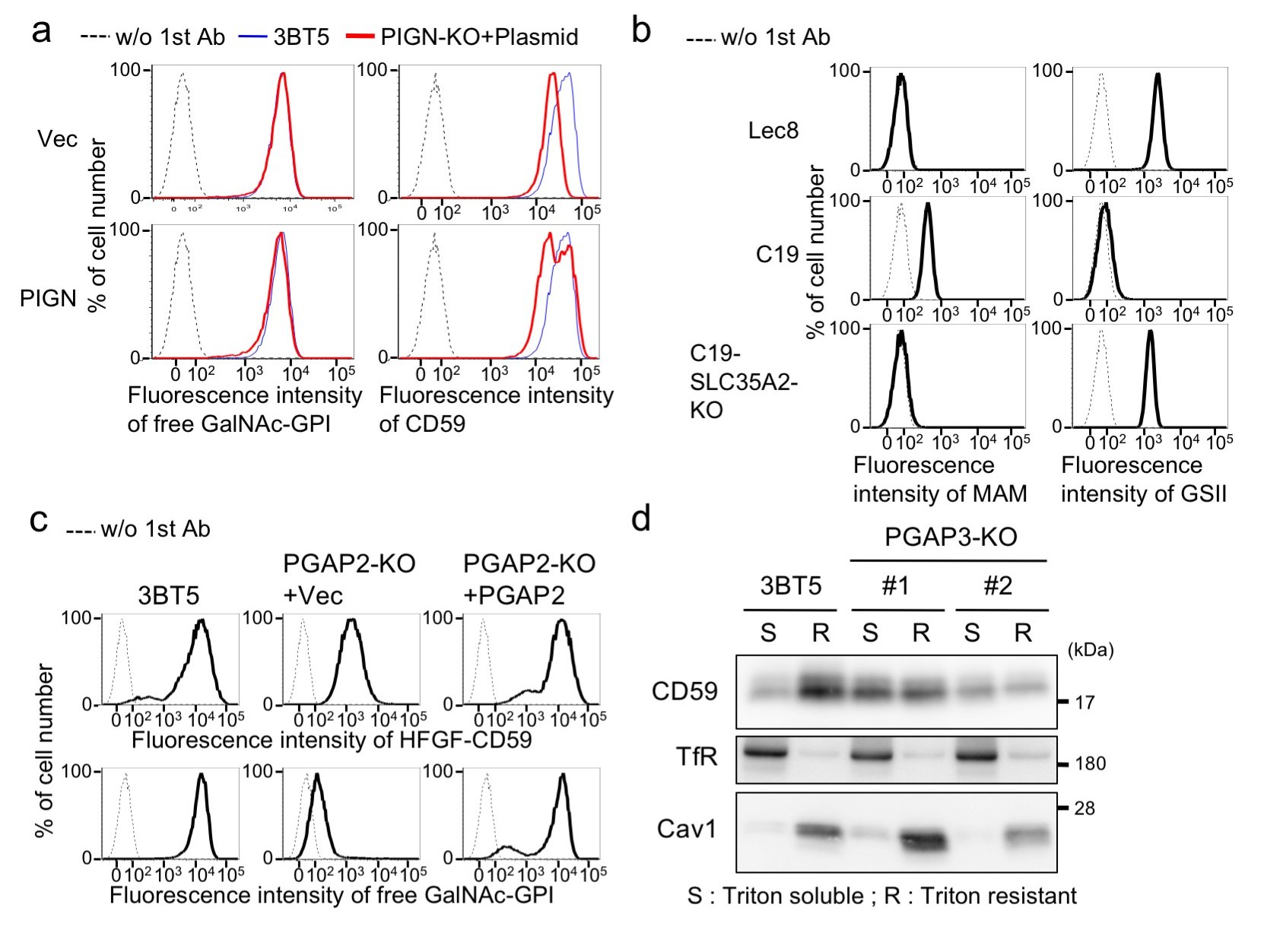


Supplementary Figure 10. Acceptor substrate specificity, timing of GalNAc modification relative to fatty acid remodeling, and T5 mAb epitope characteristics, related to Fig. 7. **a** Rescue experiment of PIGN-KO cells. 3BT5-PIGN-KO cells were transiently transfected with pME-3HA-hPIGN. Surface expression of CD59 and the staining with T5 mAb were examined. Blue, red, and dotted lines indicate 3BT5, transfected PIGN-KO cells, and background staining, respectively. Goat polyclonal anti-mIgM was used as a secondary antibody for T5 staining. Representative data from two independent experiments are shown. **b** Validation of KO of SLC35A2 in PGAP5 mutant cells (C19 cells). Cells were stained with MAM (binding to sialic acid) and GSII (binding to non-reducing terminal GlcNAc) lectins. Parental C19 cells were MAM-staining positive and GSII-staining negative whereas C19-SLC35A2-KO cells were opposite similar to Lec8 cells. Representative data from two independent experiments are shown. **c** Validation of KO of PGAP2 in 3BT5+HFGF-CD59 cells. pME-ratPGAP2 was transiently transfected into 3BT5+HFGF-CD59-PGAP2-KO cells. Surface expression of both HFGF-CD59 and free GalNAc-GPI were significantly decreased in PGAP2-KO cells, which was restored by PGAP2 expression. **d** Validation of KO of PGAP3 in 3BT5 cells by DRM separation experiment. CD59 from 3BT5 cells was mainly distributed in Triton resistant (R) fraction whereas CD59 from both 3BT5-PGAP3-KO cells were redistributed to Triton soluble (S) fractions. TfR and Cav1, markers for Triton soluble and Triton resistant fractions, respectively.


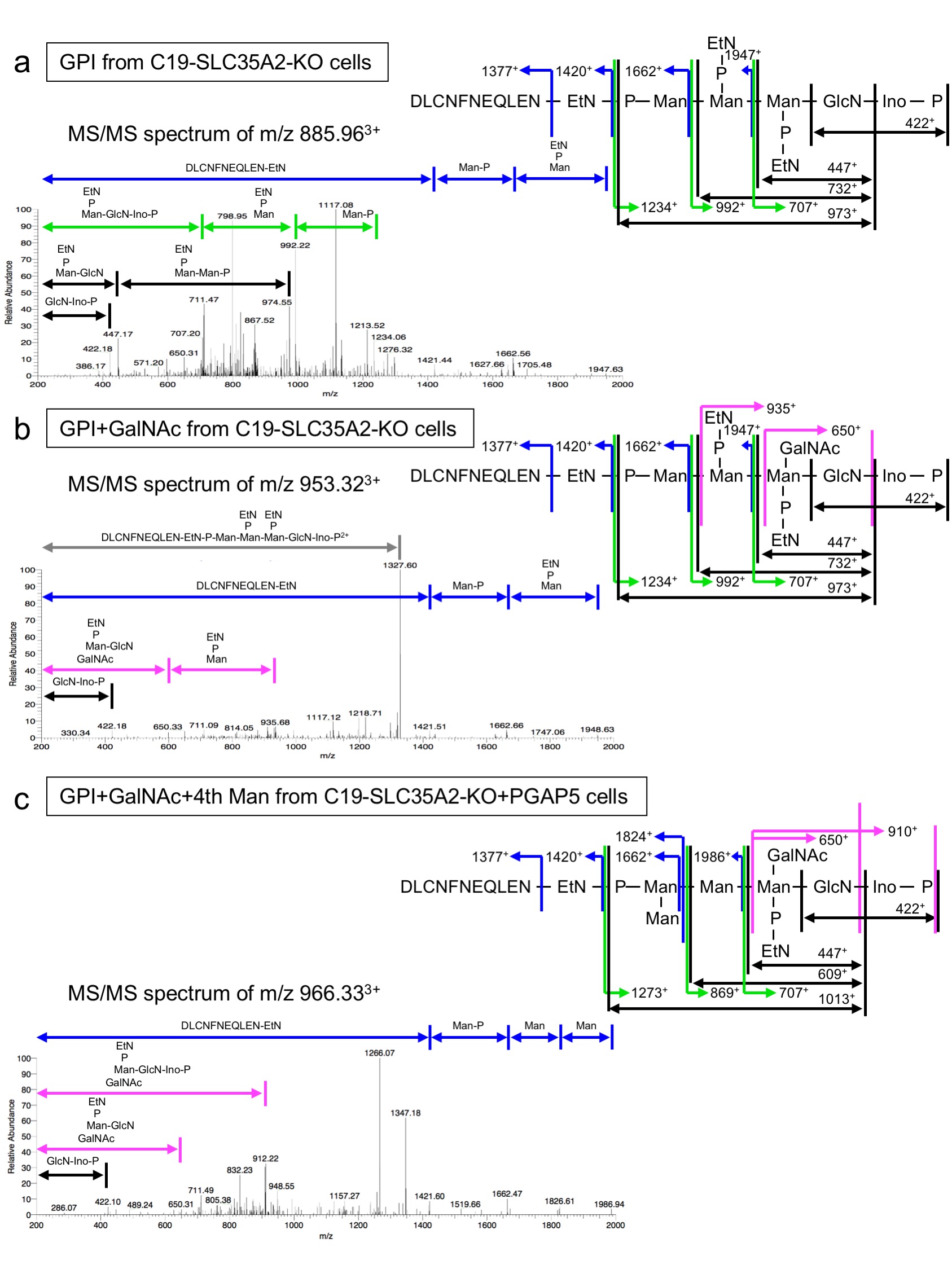


Supplementary Figure 11. ESI-MS/MS analysis of GPI structure of CD59 purified from PGAP5 mutant cells, related to Fig. 7c. **a-c** MS/MS spectra of C-terminal peptide with GPI cleaved by PI-PLC are shown. **a**, 885.96^3+^ for GPI core only from C19-SLC35A2-KO cells; **b**, 953.32^3+^ for GPI core + GalNAc from C19-SLC35A2-KO mutant cells; **c**, 966.33^3+^ for GPI core + GalNAc + 4th Man from C19-SLC35A2-KO + PGAP5 cells.


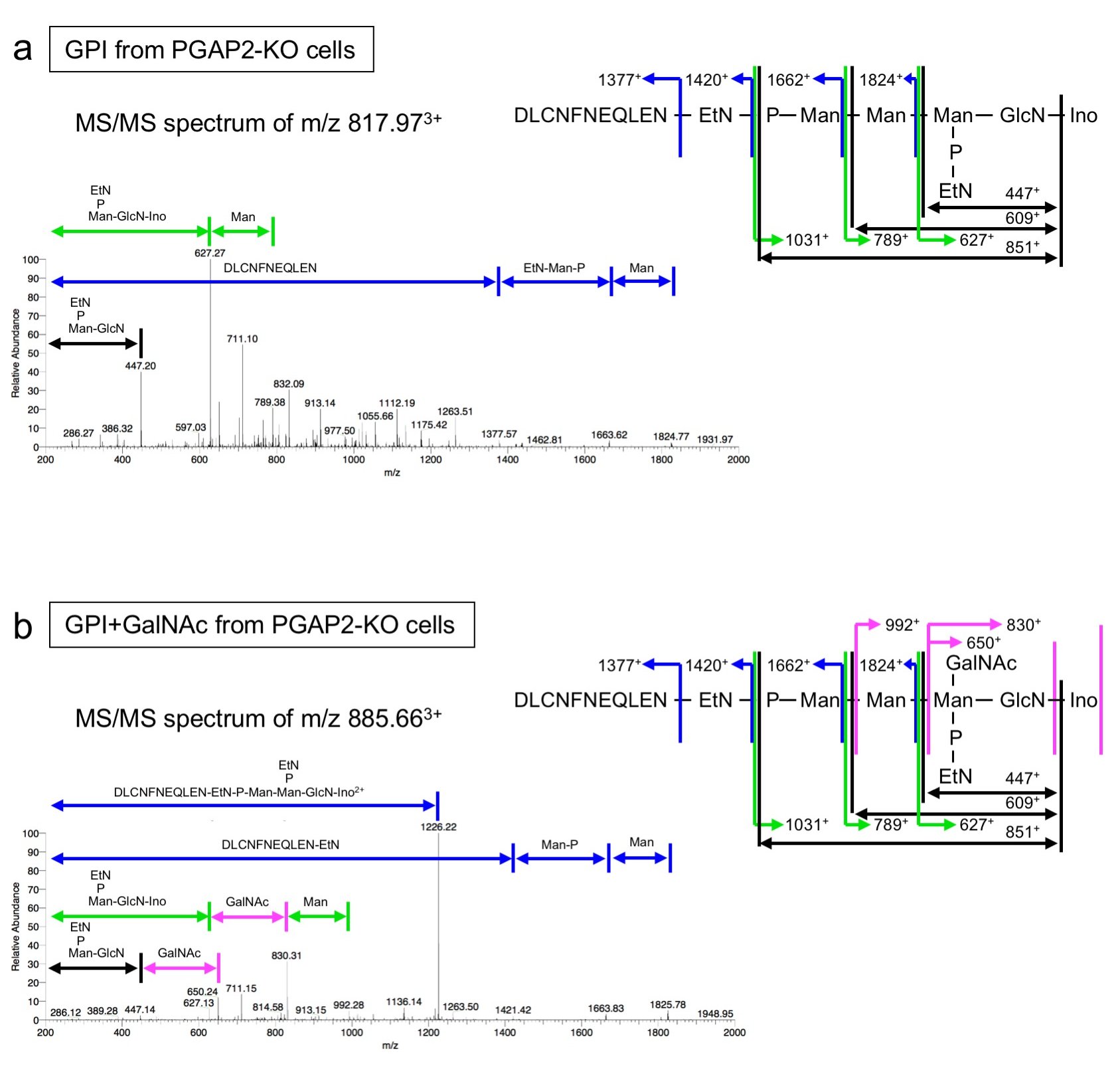


Supplementary Figure 12. ESI-MS/MS analysis of GPI structure of CD59 purified from culture medium of PGAP2-KO cells, related to Fig. 7d. **a**-**b** MS/MS spectra of C-terminal peptide with GPI cleaved by phospholipase D are shown. Because HFGF-CD59 was recovered from culture medium, in which GPI-APs are shed by GPI specific phospholipase D, detected fragments contained no phosphatidic acid. **a**, 817.97^3+^ for GPI core only from PGAP2-KO cells; **b**, 885.66^3+^ for GPI core + GalNAc from PGAP2-KO cells.


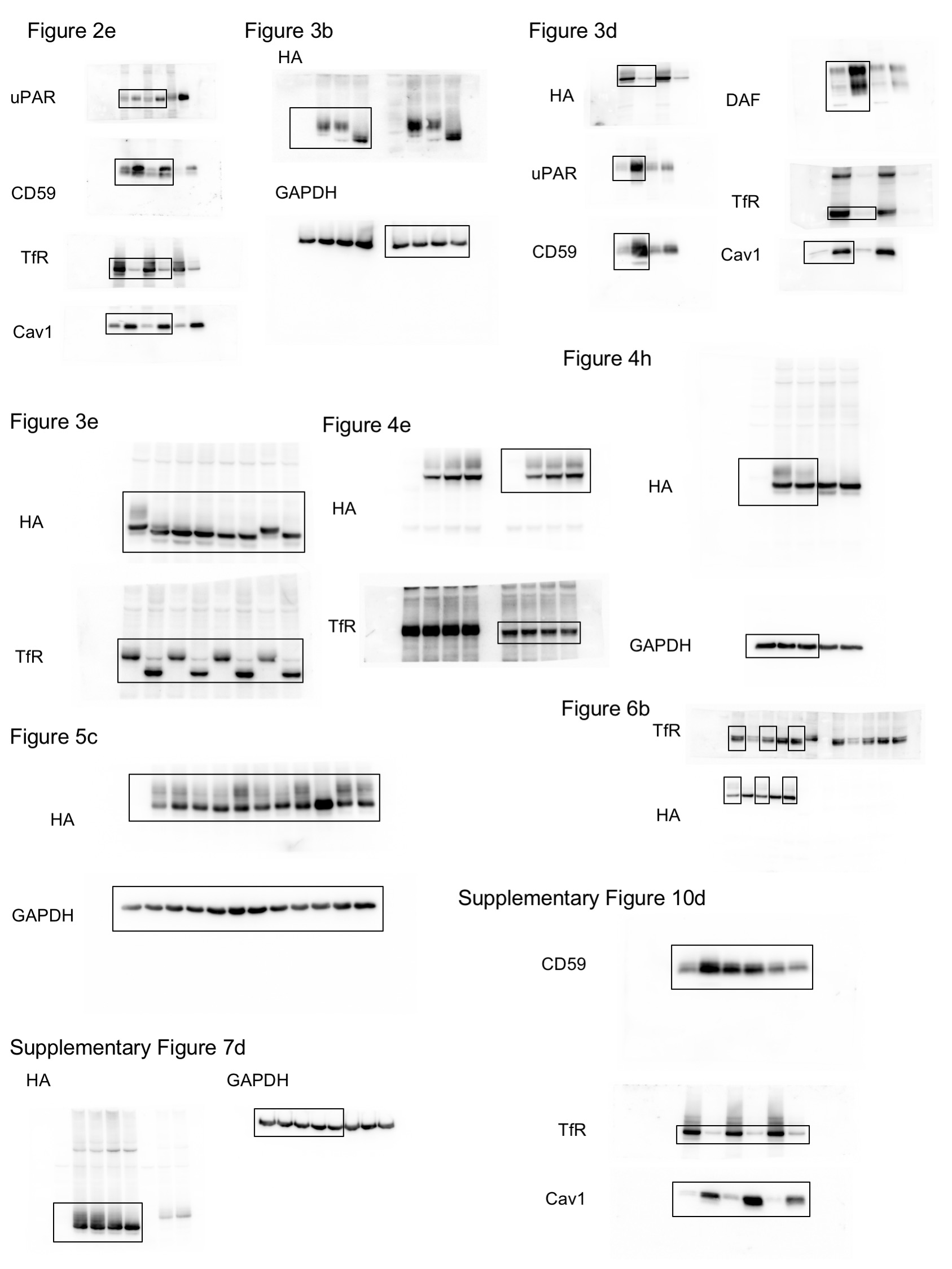


Supplementary Figure 13. Raw blots. For western blotting, a PVDF membrane corresponding to one SDS-PAGE gel was cut into pieces according to molecular size ranges of proteins of interest. The membrane pieces were incubated with relevant antibodies. Areas shown in main figures are indicated by rectangles.

Supplementary Table 1. Oligonucleotides used in this paper, related to Fig. 2, 3, 4, 5, 6, and 7.

|  | Number | Name | Sequence (5' -> 3') |
| --- | --- | --- | --- |
| gRNAs | 1 | hamPGAP4#1-Fw | caccGGAGGCTTCGGCGACTCTCC |
|  | 2 | hamPGAP4#1-Rv | aaacGGAGAGTCGCCGAAGCCTCC |
|  | 3 | hamSLC35A2#2-Fw | caccGTCATGGCTGAAGTGCTTAA |
|  | 4 | hamSLC35A2#2-Rv | aaacTTAAGCACTTCAGCCATGAC |
|  | 5 | hamPIGN#1-Fw | caccGGAGTCATTCCATGCACCAA |
|  | 6 | hamPIGN#1-Rv | aaacTTGGTGCATGGAATGACTCC |
|  | 7 | hamPIGN#2-Fw | caccGTGCCAACAGAATCTCGGCC |
|  | 8 | hamPIGN#2-Rv | aaacGGCCGAGATTCTGTTGGCAC |
|  | 9 | hamPGAP3#1-Fw | caccGCTGCTGCTAACCGTGACAG |
|  | 10 | hamPGAP3#1-Rv | aaacCTGTCACGGTTAGCAGCAGC |
|  | 11 | hamPGAP3#2-Fw | caccGCGTGCTCCGGTGCGAAGAA |
|  | 12 | hamPGAP3#2-Rv | aaacTTCTTCGCACCGGAGCACGC |
|  | 13 | hamPGAP2#1-Fw | caccGTACCAAGTTCCACTCACGC |
|  | 14 | hamPGAP2#1-Rv | aaacGCGTGAGTGGAACTTGGTAC |
| Plasmid construction | 15 | hPGAP4-EcoRI-SalI-Fw | AAAAgaattcgtcgaccaccATGAGCACTTCAACCTCTCCAG |
|  | 16 | hPGAP4-NotI-Rv | AAAAgcggccgcTAGAGGAGACTGGGATGAAAGTTG |
|  | 17 | hPGAP4(no stop)-MluI-Rv | AAAAacgcgtGAGGAGACTGGGATGAAAGTTG |
|  | 18 | hPGAP4-SalI-Fw | AAAAgtcgacATGAGCACTTCAACCTCTCCAG |
|  | 19 | 3HA-NotI-Rv | AAAAgcggccgcTTAGGCGCCAGCGTAATC |
|  | 20 | hPGAP4-delN-TM-InFusion-PstI-Fw | ATTCAGGTCATAGCctgcagTGTCACCGACTTCTACACTCTTAC |
|  | 21 | 3HA-NotI-InFusion-Rv | ACTAGTCTAgcggccgcTTAGGCGCCAGCGTAATC |
|  | 22 | hPGAP4-N-TM-MluI-Rv | AAAAACGCGTGGCCAGGGGGGCCAGCA |
|  | 23 | hPGAP4(N87A)-Fw | GAGGAGCTTCCCTCTGCCGCTGGCTCAGTGCCCATTG |
|  | 24 | hPGAP4(N87A)-Rv | CAATGGGCACTGAGCCAGCGGCAGAGGGAAGCTCCTC |
|  | 25 | hPGAP4(F283N)-Fw | CTAACCTGGATATACATGAGGAATGCCAGCCGCCCAGG |
|  | 26 | hPGAP4(F283N)-Rv | CCTGGGCGGCTGGCATTCCTCATGTATATCCAGGTTAG |
|  | 27 | hPGAP4(T347N)-Fw | CCTGCGGCCCGCCGGAACCTCACCTACCTGTCCC |
|  | 28 | hPGAP4(T347N)-Rv | GGGACAGGTAGGTGAGGTTCCGGCGGGCCGCAGG |
|  | 29 | hPGAP4(E211A)-Fw | GACTACGTCCTGATGGTAGCAGACGATGCTGTACCAGAAG |
|  | 30 | hPGAP4(E211A)-Rv | CTTCTGGTACAGCATCGTCTGCTACCATCAGGACGTAGTC |
|  | 31 | hPGAP4(D213A)-Fw | GTCCTGATGGTAGAAGACGCTGCTGTACCAGAAGAGCAG |
|  | 32 | hPGAP4(D213A)-Rv | CTGCTCTTCTGGTACAGCAGCGTCTTCTACCATCAGGAC |
|  | 33 | hPGAP4(Q26K)-Fw | GGGCAGCACTGCTGTCAAGCTCTTCATCCTAACAGTG |
|  | 34 | hPGAP4(Q26K)-Rv | CACTGTTAGGATGAAGAGCTTGACAGCAGTGCTGCCC |
|  | 35 | hPGAP4(D363A)-Fw | AGGGCTTTGGCAAGGCCATGGCACTGTACTCGC |
|  | 36 | hPGAP4(D363A)-Rv | GCGAGTACAGTGCCATGGCCTTGCCAAAGCCCT |
|  | 37 | hPGAP4(E249A)-Fw | AAGCTGTATCACCCCGCGAGGCTCCAGCACTAC |
|  | 38 | hPGAP4(E249A)-Rv | GTAGTGCTGGAGCCTCGCGGGGTGATACAGCTT |
|  | 39 | hPGAP4(H311A)-Fw | GGAGCTGGTGGGTCGGGCCTATTTCCTGGAACTGCGG |
|  | 40 | hPGAP4(H311A)-Rv | CCGCAGTTCCAGGAAATAGGCCCGACCCACCAGCTCC |
|  | 41 | hPGAP4(K362A)-Fw | GCCACAAGGGCTTTGGCGCGGACATGGCACTGTACTCG |
|  | 42 | hPGAP4(K362A)-Rv | CGAGTACAGTGCCATGTCCGCGCCAAAGCCCTTGTGGC |
|  | 43 | hPGAP4(M260A)-Fw | CTACATCAATCCAGAGCCCGCGCGGATCCTGGAATGG |
|  | 44 | hPGAP4(M260A)-Rv | CCATTCCAGGATCCGCGCGGGCTCTGGATTGATGTAG |
|  | 45 | hPGAP4(M270A)-Fw | AATGGGTTGGTGTAGGCGCGTTGCTGGGGCCCTTA |
|  | 46 | hPGAP4(M270A)-Rv | TAAGGGCCCCAGCAACGCGCCTACACCAACCCATT |
|  | 47 | hPGAP4(M302A)-Fw | CTCTTCTTCTCCCTGTATAGCGCGGGTCTGGTGGAGCTG |
|  | 48 | hPGAP4(M302A)-Rv | CAGCTCCACCAGACCCGCGCTATACAGGGAGAAGAAGAG |
|  | 49 | hPGAP4(P335A)-Fw | CCTCTCAGTGTTGCACCGCAGCCATGCTCTTCC |
|  | 50 | hPGAP4(P335A)-Rv | GGAAGAGCATGGCTGCGGTGCAACACTGAGAGG |
|  | 51 | hPGAP4(R317A)-Fw | GCACTATTTCCTGGAACTGGCGCGGCTGAGTCCTTCC |
|  | 52 | hPGAP4(R317A)-Rv | GGAAGGACTCAGCCGCGCCAGTTCCAGGAAATAGTGC |
|  | 53 | hPGAP4(T334A)-Fw | TGCCTCTCAGTGTTGCGCCCCAGCCATGCTCT |
|  | 54 | hPGAP4(T334A)-Rv | AGAGCATGGCTGGGGCGCAACACTGAGAGGCA |
|  | 55 | hPGAP4(V109A)-Fw | TGATCACCATCATCACTGCGGACAGGCAGCCTGG |
|  | 56 | hPGAP4(V109A)-Rv | CCAGGCTGCCTGTCCGCAGTGATGATGGTGATCA |
|  | 57 | hPGAP4(H247A)-Fw | GCCCTTTATCTCAAGCTGTATGCCCCCGAGAGGCTCCAG |
|  | 58 | hPGAP4(H247A)-Rv | CTGGAGCCTCTCGGGGGCATACAGCTTGAGATAAAGGGC |
|  | 59 | hPGAP4(F313A)-Fw | CTGGTGGGTCGGCACTATGCCCTGGAACTGCGGCG |
|  | 60 | hPGAP4(F313A)-Rv | CGCCGCAGTTCCAGGGCATAGTGCCGACCCACCAG |
| Genotype | 61 | hamSLC35A2-Fw1 | ATGGCAGCGGTTGGGG |
|  | 62 | hamSLC35A2-Rv1 | CTACGAACCCTTCACCTTGGTG |

Supplementary Table 2. ESI-MS/MS analysis of GPI in CD59 derived from PGAP4KO cells^1)^, related to Fig. 2c.

| Fragment | MW | Charge | m/z | % | | | | | |
| --- | --- | --- | --- | --- | --- | --- | --- | --- | --- |
|  |  |  |  | 1st experiment | | | 2nd experiment | | |
|  |  |  |  | WT | PGAP4KO  +Vec | PGAP4  KO  +PGAP4 | WT | PGAP4  KO  +Vec | PGAP4  KO  +PGAP4 |
| DLCNFNEQLEN-GPI core | 2529.90 | 3+ | 844.27 | 0 | 0 | 0 | 6.5 | 32.6 | 0 |
|  |  | 2+ | 1265.95^a^ | 0 | 0 | 9.3 | 1.6 | 31.2 | 0 |
| KDLCNFNEQLEN-GPI core | 2657.93 | 3+ | 886.98 | 20.1 | 91.4 | 1.6 | 6.9 | 29.7 | 0 |
|  |  | 2+ | 1329.97 | 0.8 | 6.6 | 0 | 0 | 4.1 | 0 |
| DLCNFNEQLEN-GPI core + GalNAc | 2732.90 | 3+ | 911.98 | 0 | 2.0 | 23.4 | 49.1 | 2.4 | 52.9 |
|  |  | 2+ | 1367.47^b^ | 0 | 0 | 0 | 10.2 | 0 | 0 |
| KDLCNFNEQLEN-GPI core + GalNAc | 2861.01 | 3+ | 954.68 | 69.3 | 0 | 65.7 | 21.5 | 0 | 47.1 |
|  |  | 2+ | 1431.50 | 0 | 0 | 0 | 0 | 0 | 0 |
| DLCNFNEQLEN-GPI core + GalNAc + Hex* | 2894.96 | 3+ | 965.99 | 9.9 | 0 | 0 | 4.2 | 0 | 0 |
|  |  | 2+ | 1448.48 | 0 | 0 | 0 | 0 | 0 | 0 |
| KDLCNFNEQLEN-GPI core + GalNAc + Hex* | 3023.06 | 4+ | 756.76 | 0 | 0 | 0 | 0 | 0 | 0 |
|  |  | 3+ | 1008.69^c^ | 0 | 0 | 0 | 0 | 0 | 0 |

^1)^GPI-containing C-terminal peptides of CD59 derived from PI-PLC-treated WT (3B2A) cells, and PGAP4-KO cells transfected with vector only or with PGAP4 cDNA.

*4th Man or Gal

^a^, ^b^, and ^c^ shown in Supplementary Fig. 3a, b, and c, respectively.

Supplementary Table 3. ESI-MS/MS analysis of GPI in CD59 derived from PGAP5-defective cells^1)^, related to Fig. 7c.

| Fragment | MW | Charge | m/z | % | | | |
| --- | --- | --- | --- | --- | --- | --- | --- |
|  |  |  |  | 1st experiment | | 2nd experiment | |
|  |  |  |  | C19+PGAP5 | C19+Vec | C19+PGAP5 | C19+Vec |
| DLCNFNEQLEN-GPI core (+ EtNP)^2)^ | 2529.90  (2652.85) | 3+ | 844.27  (885.29)^a^ | 6.2 | 2.1 | 14.8 | 2.0 |
|  |  | 2+ | 1265.95  (1327.43) | 0 | 0 | 0 | 0 |
| KDLCNFNEQLEN-GPI core (+ EtNP)^2)^ | 2657.93  (2780.94) | 3+ | 886.98  (927.99) | 2.3 | 0.4 | 1.9 | 0.2 |
|  |  | 2+ | 1329.97  (1391.48) | 0 | 0 | 0 | 0 |
| DLCNFNEQLEN-GPI core (+ EtNP)^2)^ + HexNAc | 2732.90  (2855.93) | 4+ | 684.24  (714.99) | 0 | 18.3 | 0 | 16.7 |
|  |  | 3+ | 911.98  (952.98)^b^ | 54.0 | 49.9 | 50.7 | 52.8 |
|  |  | 2+ | 1367.47  (1428.97) | 6.0 | 0 | 5.2 | 0 |
| KDLCNFNEQLEN-GPI core (+ EtNP)^2)^ + HexNAc | 2861.01  (2984.02) | 4+ | 716.26  (747.01) | 0 | 11.9 | 0 | 10.3 |
|  |  | 3+ | 954.68  (995.68) | 28.4 | 17.4 | 23.7 | 18.0 |
|  |  | 2+ | 1431.50  (1493.02) | 0.1 | 0 | 0.2 | 0 |
| DLCNFNEQLEN-GPI core (+ EtNP)^2)^ + HexNAc + Hex* | 2894.96  (3017.98) | 3+ | 965.99^c^  (1007.00) | 2.9 | 0 | 3.4 | 0 |
|  |  | 4+ | 724.75  (755.50) | 0 | 0 | 0 | 0 |
| KDLCNFNEQLEN-GPI core (+ EtNP)^2)^ + HexNAc + Hex* | 3023.06  (3146.08) | 3+ | 1008.69  (1049.70) | 0 | 0 | 0 | 0 |
|  |  | 4+ | 756.76  (787.53) | 0 | 0 | 0 | 0 |

^1)^GPI-containing C-terminal peptides of CD59 derived from PI-PLC-treated PGAP5-defective cells transfected with vector only (C19+Vec) or with PGAP5 cDNA (C19+PGAP5).

^2)^GPI core with EtNP linked to the 2nd Man was detected from C19+Vec instead of GPI core only because of PGAP5 deficiency.

*4th Man or Gal

^a^, ^b^, and ^c^ shown in Supplementary Fig. 11a, b, and c, respectively.

Supplementary Table 4. ESI-MS/MS analysis of GPI in CD59 derived from PGAP2-defective cells ^1)^, related to Fig. 7d.

| Fragment | MW | Charge | m/z | % | | | |
| --- | --- | --- | --- | --- | --- | --- | --- |
|  |  |  |  | 1st experiment | | 2nd experiment | |
|  |  |  |  | WT (3BT5) | PGAP2-KO | WT (3BT5) | PGAP2-KO |
| DLCNFNEQLEN-GPI core (- P)^2)^ | 2529.90  (2449.86) | 3+ | 844.27  (817.63)^a^ | 1.4 | 21.6 | 2.6 | 22.0 |
|  |  | 2+ | 1265.95  (1225.94) | 0 | 4.1 | 0 | 3.5 |
| KDLCNFNEQLEN-GPI core (- P)^2)^ | 2657.93  (2577.96) | 3+ | 886.98  (860.32) | 2.7 | 0.02 | 2.5 | 0.01 |
|  |  | 2+ | 1329.97  (1289.99) | 0 | 0 | 0 | 0 |
| DLCNFNEQLEN-GPI core (- P)^2)^ + HexNAc | 2732.90  (2652.94) | 3+ | 911.98  (885.32)^b^ | 52.4 | 52.1 | 53.9 | 54.0 |
|  |  | 2+ | 1367.47  (1327.48) | 1.5 | 5.0 | 2.4 | 4.9 |
| KDLCNFNEQLEN-GPI core (- P)^2)^ + HexNAc | 2861.01  (2781.04) | 4+ | 716.26  (696.27) | 0 | 1.5 | 0 | 1.3 |
|  |  | 3+ | 954.68  (928.02) | 41.7 | 15.3 | 38.5 | 13.9 |
|  |  | 2+ | 1431.50  (1391.53) | 0.3 | 0.2 | 0.2 | 0.3 |
| DLCNFNEQLEN-GPI core (- P)^2)^ + HexNAc + Hex* | 2894.96  (2815.00) | 3+ | 965.99  (939.33) | 0 | 0 | 0 | 0 |
|  |  | 2+ | 1448.48  (1408.50) | 0 | 0 | 0 | 0 |
| KDLCNFNEQLEN-GPI core (- P)^2)^ + HexNAc + Hex* | 3023.06  (2943.09) | 3+ | 1008.69  (982.04) | 0 | 0.2 | 0 | 0.1 |

^1)^GPI-containing C-terminal peptides of CD59 derived from PI-PLC-treated WT (3BT5) cells or from culture medium of PGAP2-KO cells.

^2)^GPI core lacking phosphate was detected from PGAP2-KO cells (PLD-cleaved form).

*4th Man or Gal

^a^ and ^b^ shown in Supplementary Fig. 12a and b, respectively.

Supplementary Table 5. ESI-MS/MS analysis of GPI in CD59 derived from PGAP3-KO cells^1)^, related to Fig. 7g.

| Fragment | MW | Charge | m/z | % | |
| --- | --- | --- | --- | --- | --- |
| DLCNFNEQLEN-GPI core | 2529.90 | 3+ | 844.27 | 3.0 | 7.9 |
|  |  | 2+ | 1265.95 | 2.2 | 2.0 |
| KDLCNFNEQLEN-GPI core | 2657.93 | 3+ | 886.98 | 1.4 | 3.5 |
|  |  | 2+ | 1329.97 | 0 | 0.4 |
| DLCNFNEQLEN-GPI core + GalNAc | 2732.90 | 3+ | 911.98 | 53.8 | 53.9 |
|  |  | 2+ | 1367.47 | 15.7 | 11.9 |
| KDLCNFNEQLEN-GPI core + GalNAc | 2861.01 | 3+ | 954.68 | 22.8 | 19.1 |
|  |  | 2+ | 1431.50 | 1.2 | 1.2 |

^1)^GPI-containing C-terminal peptides of CD59 derived from PI-PLC-treated WT (3BT5) cells and PGAP3-KO cells.

Supplementary Table 6. Determination of disulfide bonds in PGAP4, related to Fig. 4c.

| DTT | Cys | Fragment | Measured  m/z | Calculated m/z | Score | *P* |
| --- | --- | --- | --- | --- | --- | --- |
| - | 43 | GLLAPLACHRLL + Trioxidation | 662.8728 | 1323.7333 | 17 | 0.021 |
|  |  | ACHRLL + Trioxidation | 380.6916 | 759.3697 | 17 | 0.018 |
|  | 132, 136 | LQQCGPQCEGHQLF + 2 Dehydro | 793.3475 | 1584.6814 | 28 | 0.0016 |
|  |  | QQCGPQCEGHQL + 2 Dehydro | 663.2718 | 1324.5289 | 18 | 0.018 |
|  |  | QQCGPQCEGHQLF + 2 Dehydro | 736.8063 | 1471.5973 | 19 | 0.012 |
|  | 144 | LCNVERSVSHF + Trioxidation | 669.8089 | 1337.6034 | 27 | 0.0019 |
|  | 144, 194 | LCNVERSVSHF + CLESSL + 2 Dehydro^a^ | 646.9731 | 1937.8975 | N. D. | N. D. |
|  |  | LCNVERSVSHF + CLESSLQTY + 2 Dehydro^a^ | 777.6962 | 2330.0671 | N. D. | N. D. |
|  | 332, 333 | SVVPASQCCTPA+ 2 Dehydro | 80.7566 | 1159.5002 | 23 | 0.0053 |
|  |  | SVVPASQCCTPAML+ 2 Dehydro +Oxidation | 710.8166 | 1419.6197 | 23 | 0.0047 |
|  |  | SVVPASQCCTPAMLFPAPA+ 2 Dehydro +Oxidation | 952.4412 | 1902.8678 | 18 | 0.015 |
|  | 356 | CHKGFGKDMALYSL + Dehydro^b^ | 784.8722 | 1567.7401 | 0 | 0.93 |
| + | 43 | GLLAPLACHRLL + Trioxidation | 662.8737 | 1323.7333 | 37 | 0.00022 |
|  | 132, 136 | HRLLQQCGPQCEGHQL + 2 Carbamidomethyl | 654.3115 | 1959.9156 | 25 | 0.0032 |
|  |  | LQQCGPQCEGHQL + 2 Carbamidomethyl | 851.3768 | 1700.7399 | 32 | 0.00056 |
|  |  | LQQCGPQCEGHQL + 2 Carbamidomethyl | 851.377 | 1700.7399 | 17 | 0.022 |
|  |  | QQCGPQCEGHQL + 2 Carbamidomethyl | 721.3007 | 1440.5869 | 32 | 0.00059 |
|  |  | QQCGPQCEGHQLF + 2 Carbamidomethyl Gln->pyro-Glu (N-term Q) | 786.3206 | 1570.6293 | 40 | 0.00011 |
|  |  | QQCGPQCEGHQLF + 2 Carbamidomethyl Gln->pyro-Glu (N-term Q) | 786.3212 | 1570.629 | 67 | 2.20E^-7^ |
|  |  | QQCGPQCEGHQLF + 2 Carbamidomethyl | 794.8347 | 1587.6559 | 14 | 0.04 |
|  |  | QQCGPQCEGHQLF + 2 Carbamidomethyl | 794.8347 | 1587.6559 | 14 | 0.044 |
|  | 144 | FLCNVERSVSHF + Carbamidomethyl | 498.9097 | 1493.7085 | 18 | 0.017 |
|  |  | FLCNVERSVSHF + Trioxidation | 669.8084 | 1337.6034 | 30 | 0.0011 |
|  |  | LCNVERSVSHF + Carbamidomethyl | 449.8866 | 1346.6401 | 25 | 0.0033 |
|  |  | LCNVERSVSHF + Carbamidomethyl | 674.327 | 1346.6401 | 45 | 3.40E^-5^ |
|  |  | CNVERSVSHF + Carbamidomethyl | 617.7845 | 1233.5561 | 23 | 0.0047 |
|  |  | CNVERSVSHF + Carbamidomethyl | 617.7852 | 1233.5561 | 24 | 0.004 |
|  | 194 | VYCLESSLQTY + Carbamidomethyl | 681.816 | 1361.6173 | 38 | 0.00014 |
|  |  | CLESSLQTY + Carbamidomethyl | 550.75 | 1099.4856 | 13 | 0.049 |
|  |  | CLESSLQTY + Carbamidomethyl | 550.7501 | 1099.4856 | 16 | 0.028 |
|  | 332, 333 | SVVPASQCCTPA + 2 Carbamidomethyl | 638.7868 | 1275.5588 | 57 | 1.90E^-6^ |
|  |  | SVVPASQCCTPAML + 2 Carbamidomethyl + Oxidation | 768.846 | 1535.6782 | 50 | 9.50E^-6^ |
|  |  | SVVPASQCCTPAMLFPAPA + 2 Carbamidomethyl + Oxidation | 1010.4656 | 2018.9264 | 15 | 0.033 |
|  |  | SVVPASQCCTPAMLFPAPA + 2 Carbamidomethyl + Oxidation | 673.9825 | 2018.9264 | 24 | 0.0037 |
|  |  | SVVPASQCCTPAMLFPAPAARRTL + 2 Carbamidomethyl + Oxidation | 873.1058 | 2616.2975 | 26 | 0.0025 |

Dehydro=Dehydrogen, N. D. =Not determined

^a^ Connected peptide was discovered by Dbond software.

^a^ Fragment was confirmed by MS/MS spectrum.
