# Supplementary material for: Identification of a Golgi GPI-N-acetylgalactosamine transferase with tandem transmembrane regions in the catalytic domain
Source: Nat Commun. 2018 Jan 26;9:405. doi: 10.1038/s41467-017-02799-0 (PMC5785973; doi:10.1038/s41467-017-02799-0)
Supplement: Supplementary file 3 — Description of Additional Supplementary Files [file 41467_2017_2799_MOESM3_ESM.pdf]

Description of Additional Supplementary Files

File Name: Supplementary Movie 1
Description: 3D view of PGAP4 structural model, related to Figures 4, 5, 6 and 7. 3D structural model of PGAP4 deleted 19 amino acids from the N-terminal, PGAP4ΔN (Δ1-19), is demonstrated by ribbon and molecular surface display models. GT-A fold of PGAP4 is split by an insertion of tandem transmembrane domains. Putative catalytic site (D363), DXD-like motif (E211-D212-D213), and potential residues (H247, E249 and F313) for binding to GPI-glycan are shown by sticks. The 3D structure model shows a cavity involved in the interaction to UDP-GalNAc. Arrows indicate the juxtamembrane concave surface for GPI binding. D363 is located at the interface between the cavity for UDP-GalNAc and the juxtamembrane concave surface. H247, E249 and F313 are on the juxtamembrane concave surface for GPI binding.

File Name: Supplementary Data 1
Description: 3D structural model of PGAP4, related to Figures 4, 5 and 6. 3D structural model of PGAP4 deleted 19 amino acids from the N-terminal, PGAP4ΔN (Δ1-19), is given in a format of pdb file.
